# Chronic Replication Problems Impact Cell Morphology and Adhesion of DNA Ligase I Defective Cells

**DOI:** 10.1371/journal.pone.0130561

**Published:** 2015-07-07

**Authors:** Paolo Cremaschi, Matteo Oliverio, Valentina Leva, Silvia Bione, Roberta Carriero, Giulia Mazzucco, Andrea Palamidessi, Giorgio Scita, Giuseppe Biamonti, Alessandra Montecucco

**Affiliations:** 1 Istituto di Genetica Molecolare, Consiglio Nazionale delle Ricerche (CNR), Pavia, Italy; 2 Dipartimento di Biologia e Biotecnologie “L. Spallanzani”, Università degli Studi di Pavia, Pavia, Italy; 3 Istituto Universitario di Studi Superiori (IUSS), Pavia, Italy; 4 IFOM, Fondazione Istituto FIRC di Oncologia Molecolare, Milano, Italy; 5 Dipartimento di Scienze della Salute, Università degli Studi di Milano, Milano, Italy; University College London, UNITED KINGDOM

## Abstract

Moderate DNA damage resulting from metabolic activities or sub-lethal doses of exogenous insults may eventually lead to cancer onset. Human 46BR.1G1 cells bear a mutation in replicative DNA ligase I (LigI) which results in low levels of replication-dependent DNA damage. This replication stress elicits a constitutive phosphorylation of the ataxia telangiectasia mutated (ATM) checkpoint kinase that fails to arrest cell cycle progression or to activate apoptosis or cell senescence. Stable transfection of wild type LigI, as in 7A3 cells, prevents DNA damage and ATM activation. Here we show that parental 46BR.1G1 and 7A3 cells differ in important features such as cell morphology, adhesion and migration. Comparison of gene expression profiles in the two cell lines detects Bio-Functional categories consistent with the morphological and migration properties of LigI deficient cells. Interestingly, ATM inhibition makes 46BR.1G1 more similar to 7A3 cells for what concerns morphology, adhesion and expression of cell-cell adhesion receptors. These observations extend the influence of the DNA damage response checkpoint pathways and unveil a role for ATM kinase activity in modulating cell biology parameters relevant to cancer progression.

## Introduction

Maintenance of genome stability is beneficial for cell survival and crucial for cancer avoidance. Not surprisingly, complex molecular machineries and pathways have evolved to efficiently detect the damage and to prevent the transmission of harmful genetic information to daughter cells. In particular, the DNA damage response (DDR) involves a transient cell cycle arrest coupled with DNA repair. Failure to properly resolve DNA damage results in apoptosis or senescence [1,2] of an individual cell with little or no harm to the organism. Selection of genomically rearranged cells that escape these barriers may lead to the onset of cancer.

One parameter relevant for the final outcome is the level of DNA damage: as a generalization, while cell senescence or apoptosis is the preferred outcome following exposure to high doses, the induction of genetically altered cells frequently occurs after exposure to doses that unlikely affect viability. As most humans are only exposed to low levels of DNA-damaging agents, either exogenous or endogenous, a consideration of the response to such low levels of damage is crucial for assessing environmental cancer risk. A great deal of studies has investigated the effects due to the exposure to exogenous sources of DNA damage. However, often DNA insults result from normal metabolism including DNA replication.

We have recently characterized a model system, based on 46BR.1G1 fibroblastoid cells, suitable to investigate the strategies used by the cells to cope with low levels of chronic DNA damage [3], a condition frequently encountered in tumors, which is compatible with cell survival and proliferation. 46BR.1G1 cells derive from a patient with a genetic syndrome characterized by drastically reduced replicative DNA ligase I (LigI) activity and impaired maturation of newly synthesized DNA [4,5]. This defect results in an increased level of endogenous single (SSBs) and double stranded DNA breaks (DSBs) accompanied by phosphorylation of H2AX histone variant (γH2AX foci) [3].

LigI expression strongly correlates with the rate of cell proliferation increasing after serum stimulation of primary fibroblasts and in response to mitogenic stimuli [6,7]. Consistently, LigI is up regulated in tumor cell lines [8,9] while a strong reduction of *LIG1* gene expression is triggered by cell confluence, serum starvation and cell differentiation [6,9,10].

The chronic replication stress induced by LigI-defect in 46BR.1G1 cells does not block cell-cycle progression and elicits a moderate activation of the checkpoint pathway identified by ATM and Chk2 (Checkpoint kinase 2) kinases [3,11]. Interestingly, the signs of a DNA damage response, including histone H2AX and Chk2 phosphorylation, are commonly found in pre-neoplastic lesions, where, unexpectedly, apoptosis was suppressed relative to the hyperplasia [12,13]. In this regard, it is worth noting that the murine model of 46BR-LigI-mutation is characterized by increased incidence of spontaneous cancers with a diverse range of epithelial tumors, particularly cutaneous adnexal tumors that are rare in mice [14]. Interestingly, 46BR.1G1 cells also show an altered expression and post-translational modification pattern of SR splicing factors, including SRSF1 [15], that control the splicing profile of several gene transcripts for proteins involved in cell proliferation and apoptosis [16–21]. This finding suggests a link between DDR activation and gene expression programs and supports the hypothesis that sub-lethal doses of DNA damage may influence cell properties relevant to tumor progression. Indeed, recent studies in normal and cancer cells suggest that also cell differentiation is under the influence of DDR programs [22].

Few years ago a large-scale proteomic analysis identified over 700 proteins that are phosphorylated in response to DNA damage on consensus sites recognized by ATM and ATR, a significant fraction of which corresponds to proteins involved in cell structure and motility [23]. The physiological consequences of these modifications, however, are largely unknown. Along the same line, we have recently reported that a few proteins involved in cytoskeleton organization are differentially expressed or post-translationally modified in LigI-deficient 46BR.1G1 cells [15] compared to normal fibroblasts or to 46BR.1G1 cells in which the DNA replication defect is rescued by the stable expression of ectopic wild-type LigI (7A3 cells), which also prevents spontaneous DSBs.

During this characterization we unexpectedly observed subtle morphological differences between 7A3 and parental LigI-deficient cells with the formers more similar to normal control fibroblasts [3]. This observation led to hypothesize that cell morphology could be under the influence of DDR programs.

In this study, we examine more in detail the potential role of chronic basal DDR activation in morphological transitions. Moreover we show that the DNA damage-initiated ATM signaling directly impacts cell morphology, adhesion and migration and affects the expression profile of cell-cell adhesive receptors encoded by the cadherins family and of focal adhesion vinculin mRNAs. All these data are confirmed by bioinformatic analysis of gene expression profiles.

## Materials and Methods

### Drugs, cell lines and cell treatments

Human SV40-transformed 46BR.1G1 fibroblasts (European Collection of Cell Cultures #CB2577) and GM847 control human fibroblasts [24] were maintained in monolayer culture in DMEM supplemented with 10% FBS, 4 mM glutamine, and 50 μg/ml gentamicin (Sigma). 46BR.1G1 derivative 7A3 and 31W expressing HUC-tagged wild type LigI were grown in complete DMEM supplemented with 300 μg/ml geneticin (Sigma) [3]. To inhibit ATM kinase activity 46BR.1G1 cells were treated with 2 mM caffeine (Sigma) or 10 μM KU-55933 (gift from Dr. Mark O’Connor, KuDOS Pharmaceuticals) for 24 h.

### Immunofluorescence

Cells grown on glass coverslips were fixed in 4% paraformaldehyde and permeabilized in PBS-0.5% Triton X-100 for 10 minutes at 4°C. Actin filaments were decorated with TRITC-conjugated phalloidin (1:600, Sigma). Nuclei were stained with 0.1 μg/ml 4’,6-diamidino-2-phenylindole (DAPI, Sigma). Conventional epifluorescence microscopy was done with Optical Microscope Olympus IX71 equipped with 63x objective. Photomicrographs were taken with digital camera Cool SNAPES (Photometrics). Data acquisition was done using the MetaMorph 7.7.5 software (Universal Imaging Corporation). Pictures were deconvolved with Media Cybernetics Autoquant X2 by the application of the Adaptive Blind deconvolution algorithm for 10 iterations. The Point Spread Function (PSF) was derived from the images analyzed.

### Time-lapse

Cells were seeded at low density in a 6-well plate (2x104 cells/well). Time-lapse imaging of cell migration was performed on a NIKON Eclipse TE2000-E inverted microscope equipped with an incubation chamber (OKOLab) for temperature and CO2 control. Movies were acquired by a Cascade II 512 (Photometrics) CCD camera controlled by Metamorph Software (Universal Imaging Corporation) using a 20x magnification objective. Tracking of cells was performed using the “Manual Tracking” plug-in distributed with ImageJ software. Data were analyzed using one-way ANOVA followed by Bonferroni's Multiple Comparison Test performed using GraphPad Prism version 5.00 for Windows (GraphPad Software, San Diego California USA).

### Wound-healing assay

Cells were seeded at a density of 3-4x105 cells/ml on each side of an Ibidi Culture-Insert for live cell analysis (Ibidi), with a 500 μm ± 50 μm separation between each side of the well, and allowed to grow for 24 h. Following removal of the insert cells were incubated in DMEM. Images were taken using a digital camera Cool SNAPES connected to an Optical Microscope Olympus IX71 using the 4x objective at insert removal (0 h) and at regular intervals of 8 h. Data acquisition was done using the MetaMorph 7.7.5 software. The image analyses were done with WimScratch platform (Wimasis Image Analysis).

### Adhesion assay

For adhesion assays cells were washed 3 times with PBS, before adding Trypsin to detach the cells. Trypsin was then neutralized with Soybean Trypsin Inhibitor and serum-containing medium. The cells were subsequently seeded in 96 well plates and after 30 min, adhered cells were fixed by adding 4% paraformaldehyde and stained with 0.1% Crystal violet in 0.2 M boric acid pH 9.0 for 10 min. After washing with dH_2_O, the Crystal violet incorporated into the cells was solubilized with acetic acid (33%) and its amount measured at 620 nm wavelength using the Microplate Reader (EZ Read 400 Biochrom). The amount of Crystal violet is directly proportional to the number of adherent cells providing a rapid, direct and quantitative measurement of cell adhesion. Data were analyzed using one-way ANOVA followed by Bonferroni's Multiple Comparison Test performed using GraphPad Prism version 5.00 for Windows (GraphPad Software, San Diego California USA).

### qRT-PCR

RNA was isolated with SV Total RNA Isolation System (Promega) following manufacturer’s instructions and reverse transcribed with Oligo d(T)16 and MuLV Reverse Transcriptase (Applied Biosystem); qRT-PCR was performed with QuantiTect SYBR Green PCR Master Mix (Qiagen) along with gene specific primers (S1 Table) on LightCycler 480 (Roche). The fold increase of cDNA level retrotranscribed from RNA was determined as follows: 2-(CT test-CT control) [25], where test refers to the gene of interest and control refers to the reference gene RPLP0. CT value indicates the cycle at which the amplified product passes the threshold. Statistical significance was determined by Students t test.

### Cell lysates and Western blotting

Cell extracts were prepared as previously described [26] and analyzed by Western blotting with the following primary antibodies: polyclonal goat anti-human cadherin-13 antibody (AF3264, R&D), 1:200; polyclonal rabbit anti-R-cadherin antibody (NBP1-90370, Novus biological), 1:300; anti-tag Muscle Actin (HUC1-1) monoclonal mouse antibody (sc-53141, Santa Cruz Biotechnology, Inc.), 1:100; polyclonal rabbit anti-Histone H2AX (phosphor S139) (γH2AX) antibody (ab81299, Abcam) 1:100000; monoclonal mouse anti α-Tubulin antibody (T9036, Sigma) 1:1000. Primary antibodies were revealed with peroxidase-conjugated Donkey anti-Goat (ab6885, Abcam), Peroxidase-conjugated AffinityPure Goat anti-Rabbit (111-035-144, Jackson Lab) and anti-Mouse (115-035-146, Jackson Lab) antibodies and enhanced chemiluminescence system (Super Signal West Pico Pierce or Luminata Crescendo/Forte Western HRP substrate Millipore).

### Microarray analysis

Whole Human Genome 4 x 44k Oligo Microarrays (Agilent Technologies) were used to compare the expression profiles of 46BR.1G1 and 7A3 cell lines. The entire procedure was described in Chikh and coworkers [27]. Briefly: equal amounts of mRNA from the two cell lines were subjected to one round of amplification by the Amino Allyl MessageAmp II aRNA kit (Ambion Inc., Austin, TX). Labeling was obtained using NHS ester Cy3 or Cy5 dyes (GE HealthCare, Buckinghamshire, UK) and hybridization was performed with dye-swap duplication. All steps were performed using the Gene Expression Hybridization kit (Agilent Technologies) according to manufacturer instructions. Slides were scanned with the dual-laser microarray scanner Agilent G2505B and images were analysed with the Feature Extraction software version 9.5 (Agilent Technologies). Agilent Feature Extraction output files were processed with the Resolver SE System (Rosetta Biosoftware, Seattle, WA). Microarray expression data were deposited at the GEO repository under the accession number: GSE56317.

### RNA-Seq analysis

Total RNAs isolated from 7A3 and 46BR.1G1 cells were subjected to polyA+ fraction selection and transformed in a cDNA library for next-generation sequencing by the use of the TruSeq RNA Sample Prep kit (Illumina) according to manufacturer’s protocol. A total of 120 million sequence reads were obtained for each cell line in three biological replicates on an Illumina HiSeq2500 instrument (40 million reads / replicate). Raw reads were subjected to standard quality control procedures with the NGSQC-toolkit software and aligned to the human genome reference sequence (NCBI37/hg19) by the TopHat alignment software [28]. Genes were annotated and quantified according to the TopHat-Cufflinks protocol and differential gene expression analysis was performed by CuffDiff [29]. RNA-Seq raw data were deposited at the NCBI Sequence Read Archive (SRA; http://www.ncbi.nlm.nih.gov/sra/) repository under the accession number: SRP058222.

### Expression profiles and literature data analysis

Gene expression data from microarrays and next-generation sequencing were analysed through the use of QIAGEN’s Ingenuity Pathway Analysis (IPA, QIAGEN Redwood City, www.qiagen.com/ingenuity). The list of proteins target of ATM/ATR was assembled from large-scale proteomic studies with the following criteria: (i) from the study of Matsuoka et al. [23] a total of 683 proteins showing an increased phosphorylation after IR damage and ATM checkpoint activation were included; (ii) from the study of Bensimon et al. [30] a total of 228 proteins whose phosphorylation state was found dependent or regulated by ATM (see Supplementary Table S11 in [30]); (iii) from the study of Bennetzen et al. [31] a total of 209 proteins whose phosphorylation change resulted significant in at least one of the observed time points (see Supplementary Table 1 in [31]). This approach resulted in the compilation of a list of 957 proteins phosphorylated on consensus sites recognized by ATM and/or ATR in response to DNA damage. This list was compared with the list of the 134 transcription factors predicted to act as upstream regulators (IPA analysis p-value<0.05) of the genes defined as differentially expressed by the microarray or the RNA-Seq analyses.

## Results

### DDR induced by LigI-deficiency accounts for some morphological features of 46BR.1G1 cells

We have previously suggested that the LigI-defect, in addition to produce replication-mediated DNA damage, is associated with a slightly different morphology of 46BR.1G1 compared to that of normal cultured fibroblasts. Interestingly, the fibroblast-like shape could be rescued by stably expressing exogenous wild type LigI (7A3 cells) [3]. Based on this qualitative observation we hypothesized that cell morphology could be a target of DNA damage and of the ATM/Chk2 checkpoint pathway.

**Fig 1:**
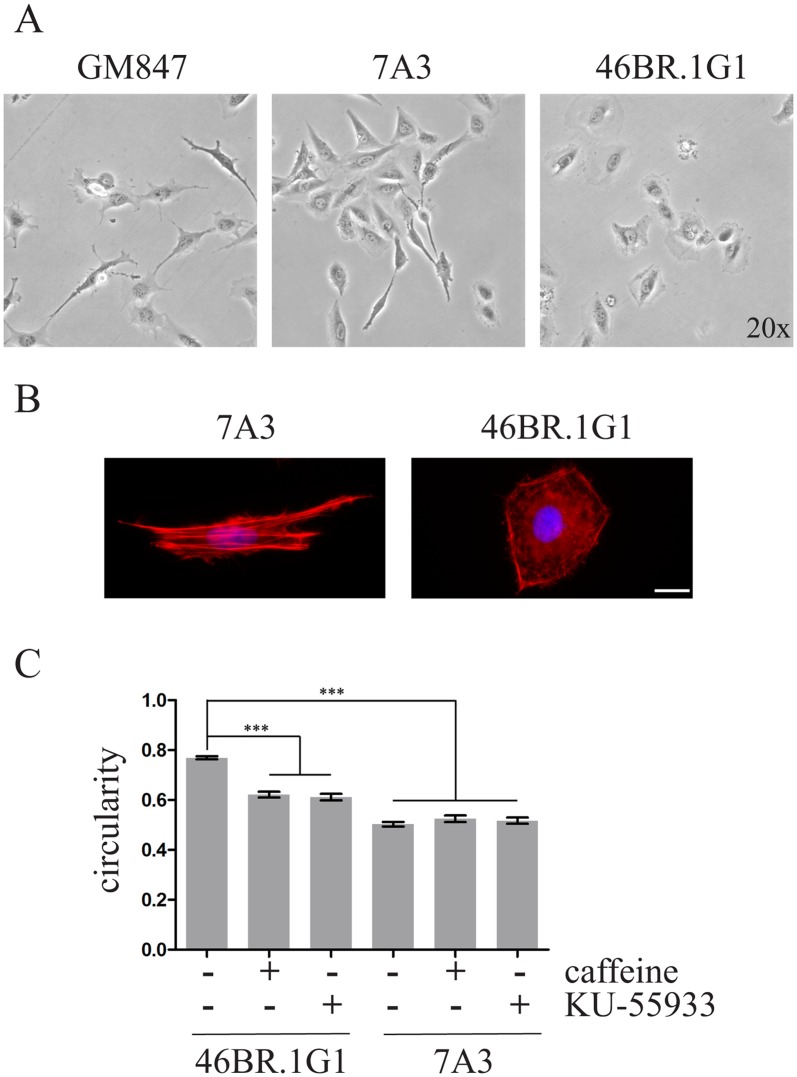
Correction of LigI defect affects cell morphology. A) Time-lapse imaging of cell migration. Cells were seeded at low density and monitored by time-lapse microscopy as described in Materials and Methods. Representative still images of control fibroblasts (GM847), complemented 7A3 expressing wild type LigI and LigI-deficient 46BR.1G1 cells are shown. B) Distribution of actin cytoskeleton. Cells were grown on coverslips and decorated with TRITC-conjugated phalloidin. Nuclei were counterstained with DAPI. C) Quantification of morphological differences between 46BR.1G1 and 7A3 cells was determined by measuring the average ratio between the short and long axes of the cell (circularity). Circularity was also measured in the presence (+) of caffeine and KU-55933 as described in Materials and Methods. At least 100 cells/conditions for each cell line were analysed. Bars show mean ± SEM. *** P < 0.001.

To more precisely characterize this aspect and to understand whether the effect on cell morphology involved the DDR, we monitored by time-lapse microscopy 46BR.1G1 and 7A3 in the presence or not of checkpoint inhibitors. We compared four different parameters: morphology, directionality, accumulated distance, and velocity. As shown in Fig 1A and S1, S2 and S3 Videos, 46BR.1G1 cells are significantly more rounded compared to 7A3 cells that express ectopic wild type (wt) LigI and show a fibroblast-like morphology. A similar difference was observed when 46BR.1G1 were compared to another independent clone (31W) expressing wt LigI (S1 Fig) confirming that the effect on cell morphology is not cell clone specific. This shape difference is accompanied by an altered distribution of the actin cytoskeleton. As expected for normal fibroblasts, 7A3 cells display long stress fibers, running along the entire length of the elongated cells. Conversely, in 46BR.1G1 actin stress fibers are mainly confined to a cortical rim, while only short actin filaments are detectable in the cytoplasm (Fig 1B).

We quantified these morphological differences by measuring the ratio between short and long axes of the cell (circularity). This analysis revealed a significant difference in the circularity index, which is 0.77 (SEM±0.005) for 46BR.1G1 and 0.50 (SEM±0.009) for 7A3 cells (Fig 1C), and provided a quantitative basis to our previous suggestion that LigI-deficiency impacts cell shape. However, despite this morphology change, none of the migration parameters measured in this assay, including cell velocity, accumulated distance and directionality, were significantly altered by LigI activity (S2 Fig).

To verify the hypothesis that morphological differences could be due to the increased basal level of DNA damage we treated 46BR.1G1 cells with the checkpoint inhibitor caffeine or the more specific ATM inhibitor KU-55933 [32]. As shown in Fig 1C, these drugs significantly reduced the circularity of 46BR.1G1 without affecting the shape of 7A3 cells. Thus, ATM activation in LigI-deficient cells is a determinant of 46BR.1G1 cell morphology, further pointing to a link between checkpoint kinases and cytoskeleton organization.

**Fig 2:**
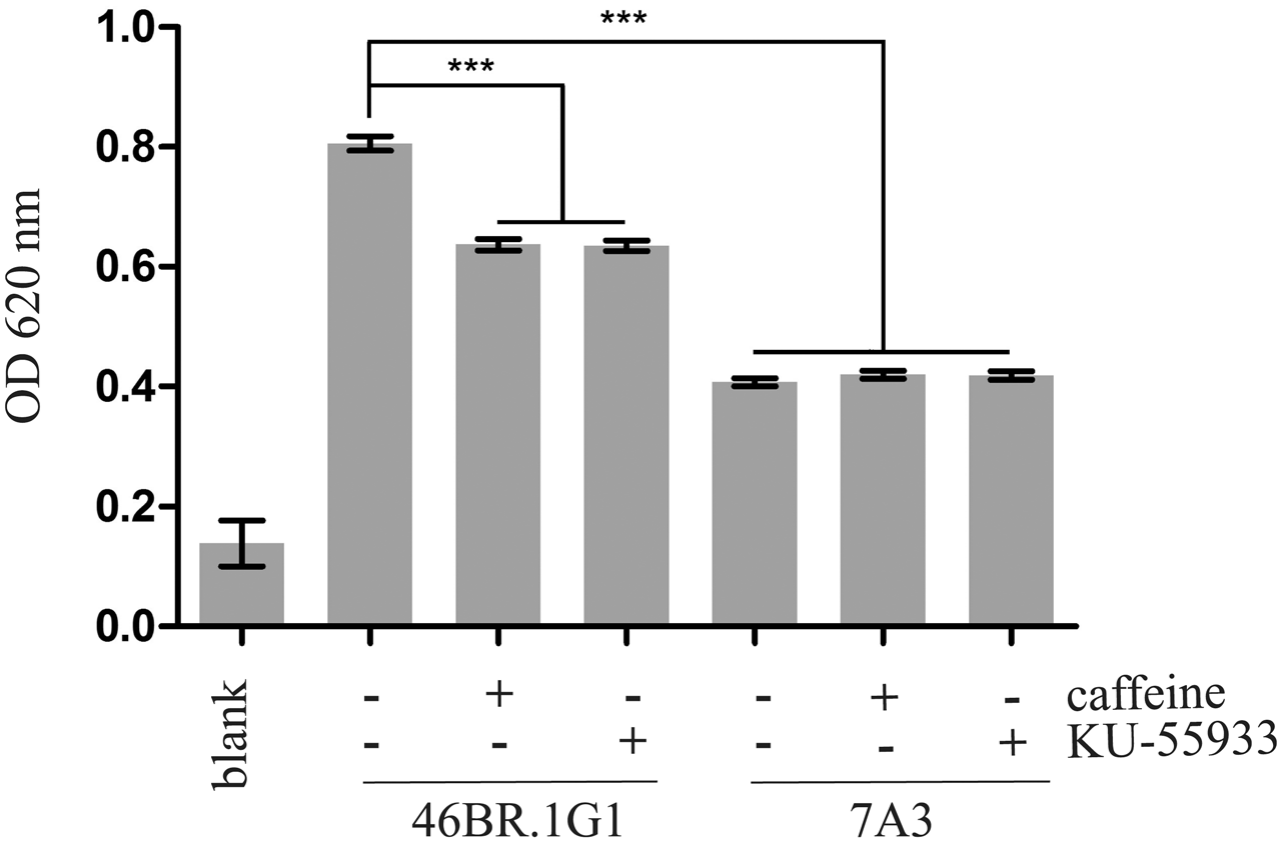
LigI-deficient 46BR.1G1 cells adhere more efficiently to the plate than complemented 7A3 cells. Cells were plated on 96-well plate and allowed to adhere for 30 minutes before fixing. Cells were stained with Crystal Violet, solubilized with acetic acid and quantified by measuring the OD at 620 nm. Data are shown as mean ± SEM of four independent experiments.

Changes in cell morphology may be linked to an altered cell adhesion. To verify this aspect, we challenged the two cell lines in a standard cell adhesion assay. As shown in Fig 2, 46BR.1G1 cells adhered more efficiently to the plate than LigI-proficient 7A3 cells. Notably, incubation with caffeine and KU-55933 significantly reduced adhesion of 46BR.1G1 but not of 7A3 cells. Altogether these results suggest that the activation of the ATM/Chk2 signaling pathway has an important role in the effect of replication stress induced by LigI-deficiency on cytoskeleton organization and cell adhesiveness.

**Fig 3:**
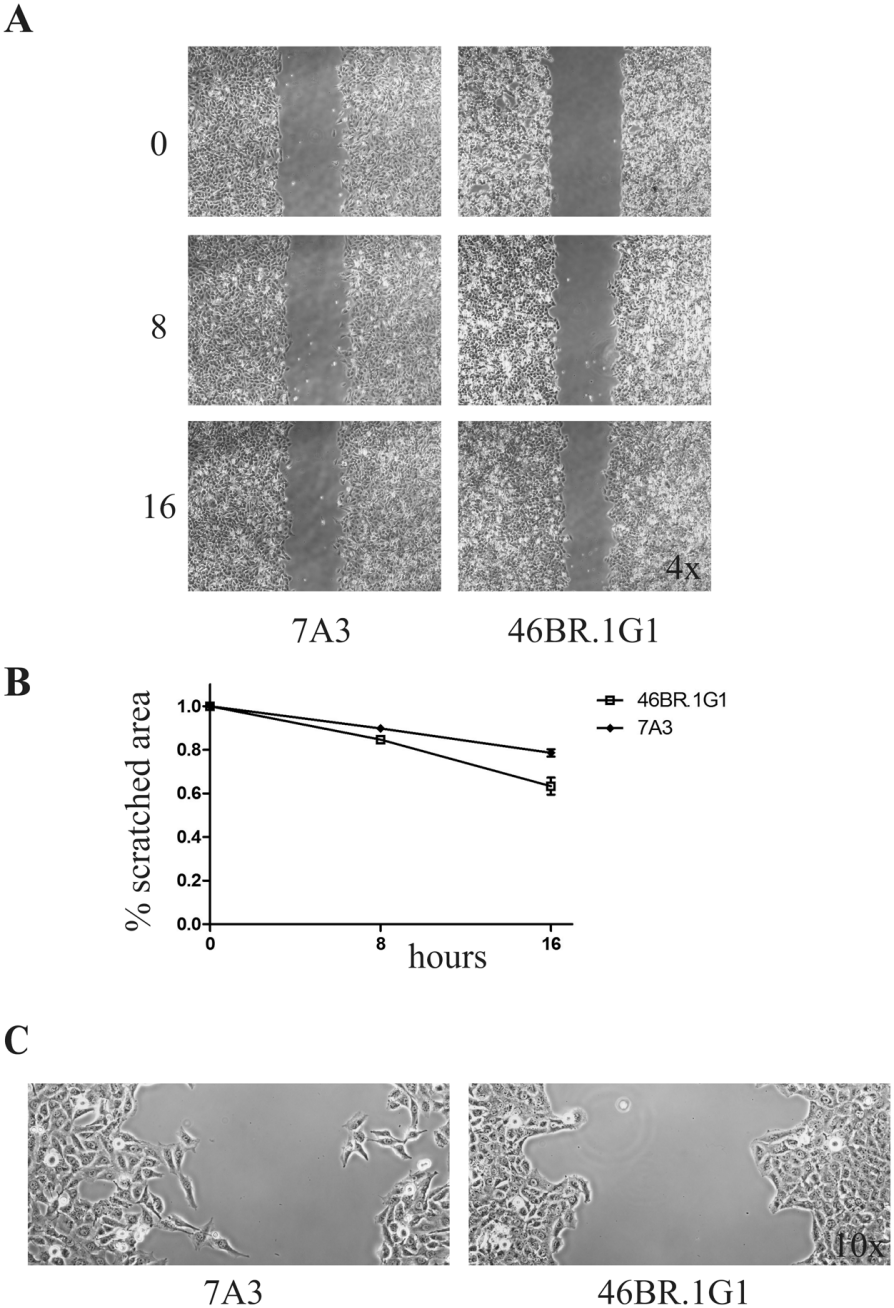
LigI-deficiency affects directional migration. A) Wound-healing assay. The same number of 46BR.1G1 and 7A3 cells were seeded in each side of an Ibidi culture insert and incubated for 24 h. Cells were photographed at the time of insert removal (0 h), 8 h and 16 h after. Magnification: 4x. B) The percentage of the scratched area at each time point was calculated with the WimScratch tool (Wimasis Image Analysis). Data are shown as mean ± SEM of three independent experiments. C) Representative images of 7A3 and 46BR.1G1 directional migration in the scratched area (magnification 10x).

Although the time-lapse experiments fail to detect differences in the random migration of individual 7A3 and 46BR.1G1 cells, it is plausible that the increased adhesion of LigI-deficient cells may affect directional migration or collective locomotion. To verify this possibility we challenged 7A3 and 46BR.1G1 cells in a wound-healing assay. Under these conditions, cells are forced to move directionally into the open wound thus adopting a polarized mode of cell locomotion. As shown in Fig 3, 46BR.1G1 cells heal the wound slightly faster than 7A3. The proliferation rate of 46BR.1G1 cells is slightly reduced with respect to 7A3 [3], ruling out that their faster migration is accounted by different rate of growth. Moreover, differently from 7A3 cells, LigI-deficient cells tend to migrate together in cohesive sheets, suggesting that the balance between cell-cell interactions and cell-plate adhesion is shifted toward the former, resulting in improved collective directionality (Fig 3C).

On the basis of these observations, we conclude that DNA damage signaling could transduce information influencing cytoskeleton organization, cell adhesion and migration, three functional parameters frequently altered in tumors.

### DDR induced by LigI-deficiency affects the expression of genes involved in cell adhesion and migration

The results described above suggest that DNA replication-dependent DNA damage caused by LigI-deficiency can induce morphological changes and affect important cell features such as cell adhesion and motility. All these events appear to depend, at least in part, on the activation of the ATM pathway, which can influence both post-translational modifications and changes in expression programs. In agreement with this hypothesis, we have previously shown that LigI-deficiency affects the phosphorylation profile of splicing regulator SRSF1 [15], which controls the splicing pattern of a number of genes in the apoptotic pathway and is necessary for cell survival [20,33].

In order to characterize the impact of LigI-deficiency on gene expression we compared total RNAs from 46BR.1G1 and 7A3 cells by the microarray technology. By this approach we identified a total of 2114 differentially expressed genes (LFC≥|1|; adjusted p-value ≤ 0.05). Interpretation of this set of genes using the IPA Core Analysis tool (Ingenuity) selected 39 categories of the Bio-Function group, corresponding to a total of 642 terms statistically enriched with a p-value < 1x10^-3^. Among the top ten categories (357 terms), six include genes involved in cell proliferation, development and survival, which may have a role in the capacity of the cells to cope with moderate replicative stress. In agreement with our previous proteomic analysis [15], the “Gene expression” category includes the splicing factor SRSF6 (already known as SRp55) gene, reinforcing the notion that splicing regulation is part of the cell response to the type of DNA damage produced by LigI deficiency. Interestingly, 3 out of the 10 most-enriched categories concern biological processes connected to the cytoskeleton (Table 1). In particular, the “Cellular Assembly and Organization” category includes 34 terms with enrichment p-values <5x10^-4^ and the “Cell Morphology” and “Cell Movement” categories include respectively 38 and 46 terms exceeding the same p-value threshold (see S2 Table). Thus, genes differentially expressed in 46BR.1G1 vs 7A3 cells are enriched in categories compatible with the biological differences evidenced by the functional assays described above.

**Table 1:** Enrichment analysis of IPA molecular function categories.

IPA Categories	46BR.1G1 vs 7A3 microarray (n = 2114)	46BR.1G1 vs 7A3 RNA-seq (n = 855)	46BR.1G1 vs 7A3 microarray and RNA-seq (n = 375)
Cellular Assembly and Organization	1.94E-18	5.63E-14	1.72E-09
Cellular Function and Maintenance	1.94E-18	5.63E-14	1.72E-09
Cell Morphology	6.23E-17	-	1.93E-10
Cellular growth and Proliferation	3.85E-16	7.72E-14	-
Cell death and Survival	7.77E-16	1.53E-15	-
Gene Expression	8.28E-14	-	-
Cellular Movement	1.40E-11	5.48E-17	5.29E-11
Connective Tissue Development and Function	1.75E-11	-	-
Organismal Survival	5.11E-11	-	-
Cellular Development	1.37E-10	5.91E-14	2.29E-09
Embryonic Development	-	9.01E-14	1.81E-09
Organismal Development	-	9.01E-14	1.81E-09
Tissue Development	-	1.22E-13	3.15E-09
Tissue Morphology	-	1.34E-12	-
Nervous System Development and Function	-	-	3.37E-10
Cell Cycle	-	-	3.28E-09

To confirm this analysis we decided to study the expression profiles in 46BR.1G1 and 7A3 cell lines by next-generation RNA sequencing. By this approach we identified a total of 855 genes differentially expressed with a LFC ≥ |1| and a q-value ≤0.05. The evaluation of the complete list by the IPA Core Analysis tool identified 46 statistically significant categories of the Bio-Function group, which include a total of 786 terms (p-value <1x10^-3^). Interestingly, 7 of the top ten categories where in common with those identified by the analysis of microarray data (Table 1). Four categories correlated with developmental processes (“Embryonic Development”, “Organismal Development”, “Tissue Development” and “Cellular Development”). Among the cytoskeleton related categories, “Cellular movement” was the most enriched one (40 terms with p-value < 5 x10^-4^) and “Cellular Assembly and Organization” was ranked 3^rd^ (19 terms with p-value < 5 x10^-4^). “Cell Morphology” was not included in the top ten list, however it was present at the 11^th^ position with 26 terms exceeding the same p-value threshold (see S2 Table). Thus, although the list of genes identified by RNA-Seq is smaller than that selected by the microarray, a strong concordance in the functional categories exists (see S3 Table for the list of the genes).

By crossing the gene lists selected by the two genome-wide approaches we identified a common set of 375 genes that were then classified in bio-functional categories using the IPA Core Analysis tool. Remarkably, a strong overlap with categories present in the microarray or RNA-Seq data (Table 1) was detectable. In particular, “Cellular movement” is the most-enriched category and contains 28 terms exceeding the threshold of p-value< 5x10^-4^ (see Table 1 and S2 Table). Interestingly most of the categories concern cell organization, movement and differentiation features.

Thus, gene expression analysis performed with two independent approaches selects bio-functions that may account for the morphological and migration properties of LigI-deficient cells.

### Expression of cadherins is affected by LigI deficiency in an ATM-dependent manner

**Fig 4:**
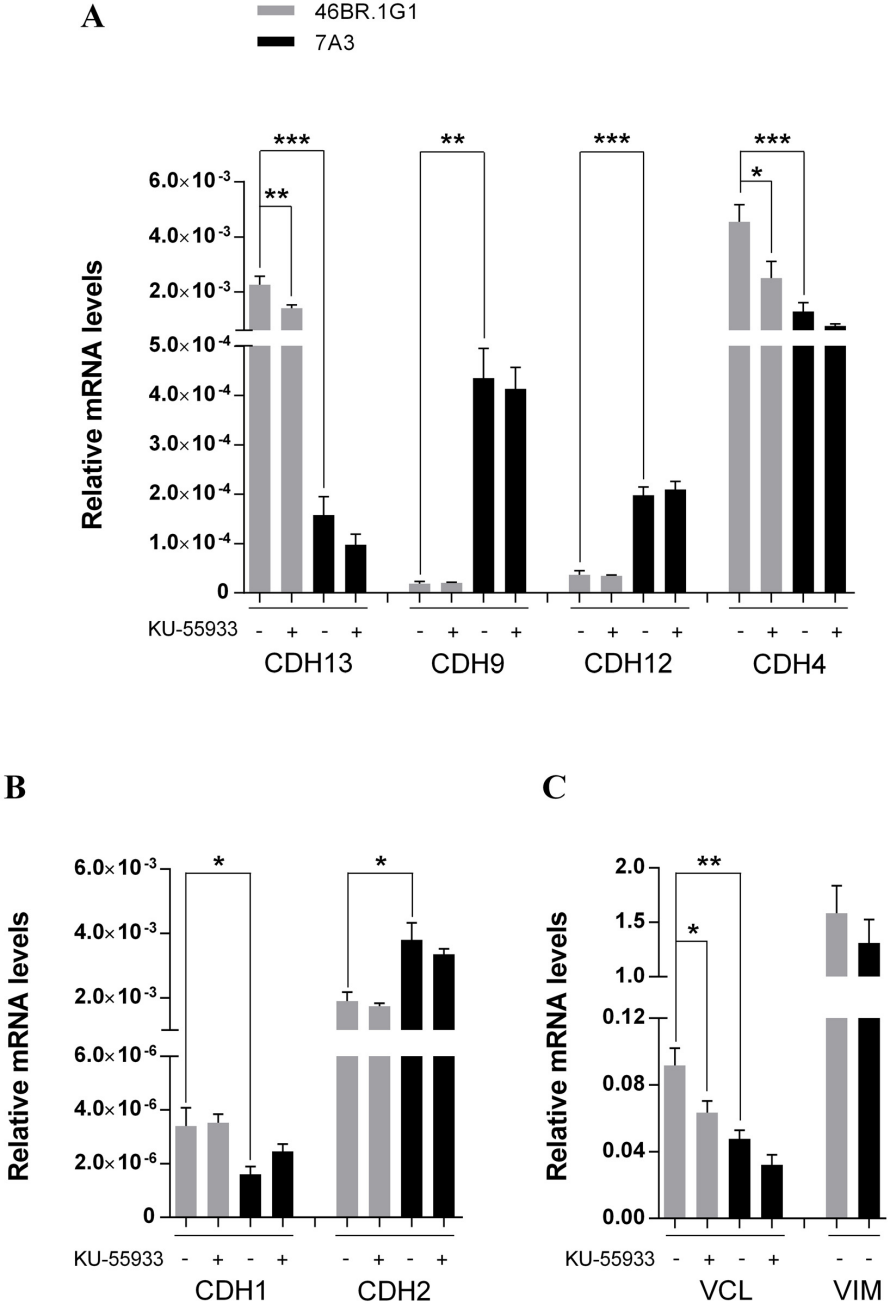
Analysis of vinculin, vimentin and cadherins gene expression by real time RT-PCR. The panels show the relative expression levels of the indicated transcripts in 46BR.lG1 (gray bars) and 7A3 cells (black bars) before (-) and after (+) incubation with 10 μM KU-55933. Gene transcripts have been internally normalized versus RPLP0 expression levels. Data are shown as mean ± SEM of four independent experiments. CDH: cadherin, VCL: vinculin, VIM: vimentin. * P < 0 .05, ** P < 0.01, *** P < 0.001.

**Fig 5:**
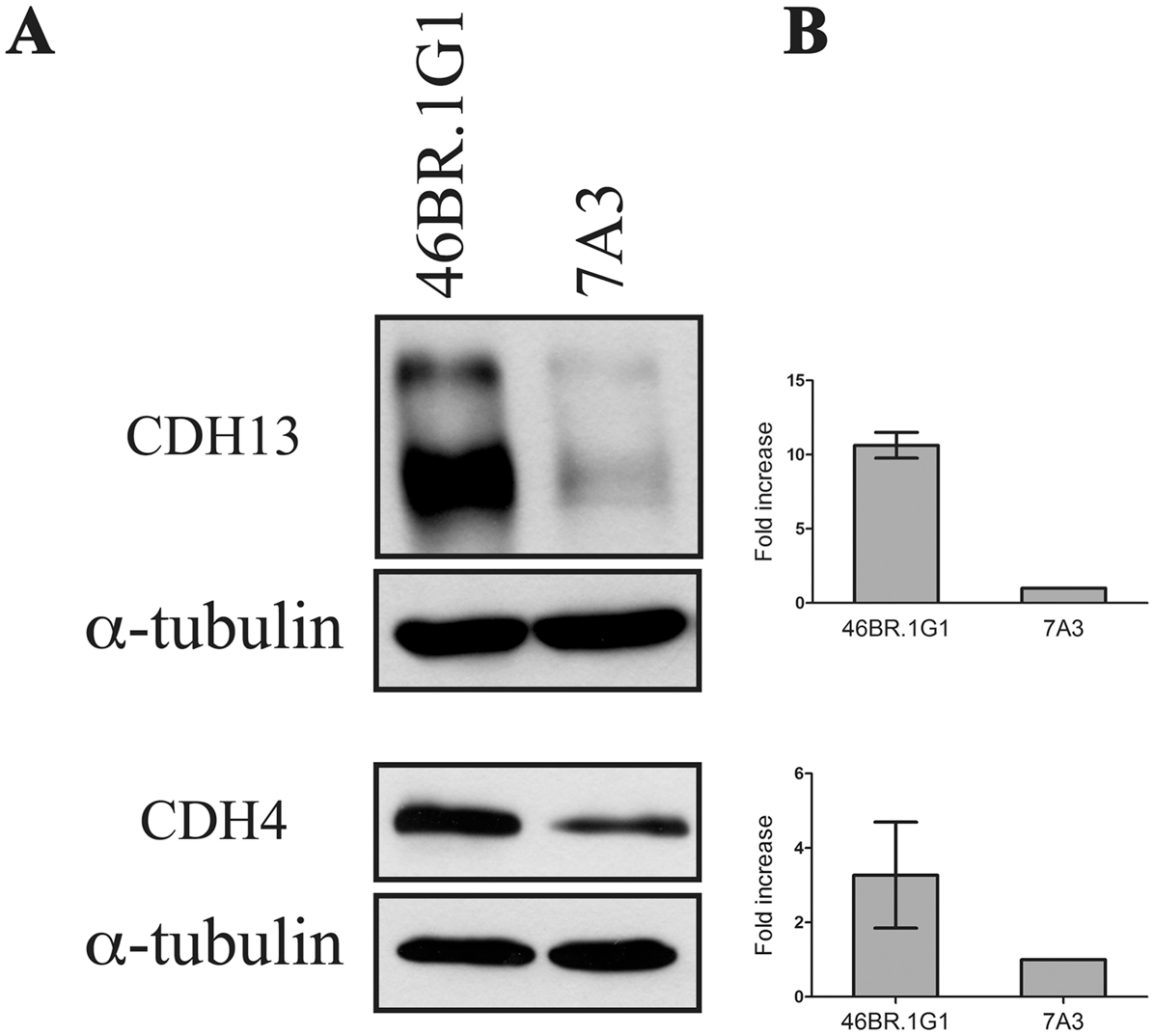
Differential expression of cadherin 13 and cadherin 4 proteins in 46BR.1G1 and 7A3 cells. (A) Cell lysates from 46BR.1G1 and 7A3 cells were analyzed by Western blotting with anti-cadherin 13, anti-cadherin 4, and anti-α-tubulin antibodies. (B) Quantification of the assay was performed by densitometric analysis with NIH ImageJ 1.43 program. Bars show mean ± SEM of three independent experiments.

**Fig 6:**
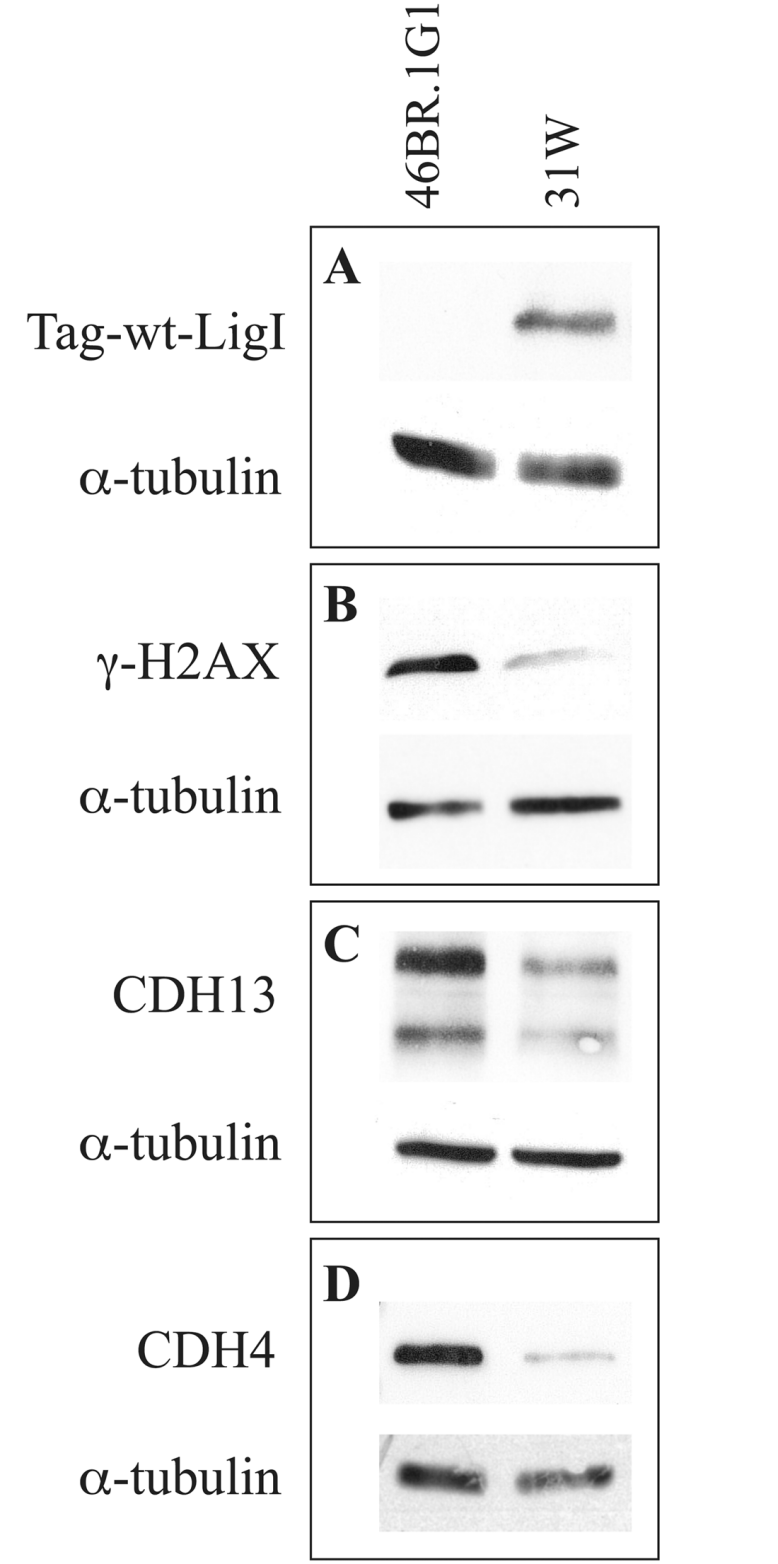
Differential expression of cadherin 13 and cadherin 4 proteins in 46BR.1G1 and 31W cells. Cell lysates from 46BR.1G1 and 31W cells were analyzed by Western blotting with antibodies against the indicated proteins.

As a further validation of the high-throughput analyses we decided to measure by qRT-PCR the expression of a few selected genes. IPA categories describing the process of cell migration include vinculin and some members of the cadherin superfamily involved in cell adhesion and migration [34]. We focused on genes of the cadherin family, some of which were detected as differentially expressed in 46BR.1G1 vs 7A3 cells by both microarray and RNA-Seq analyses. As shown in Fig 4, in agreement with the genome wide analyses, qRT-PCR measured statistically significant differences in the expression of cadherin 4 (*CDH4* also called R-cadherin), cadherin 13 (*CDH13*, H-cadherin), cadherin 9 (*CDH9*, T1-cadherin) and cadherin 12 (*CDH12*, N-Cadherin 2). Notably CDH4 is a critical regulator of epithelial phenotype [35] and CDH13 levels are frequently down regulated in invasive carcinoma cells [36]. In order to verify the effect of this regulation at the protein level, cell extracts from 46BR.1G1 and 7A3 cells were immunoblotted with antibodies against CDH13 and CDH4, whose transcripts are overexpressed in LigI-deficient cells. Fig 5 shows that, in agreement with the qPCR analysis, both proteins are overexpressed in 46BR.1G1 cells. The down-regulation of CDH13 and CDH4 in LigI-proficient cells was also confirmed in 31W cells (Fig 6) ruling out the possibility that the observed change in gene expression was cell clone specific. Notably, the differential expression of these cadherins is consistent with the idea that LigI-deficiency may induce a shift toward an epithelial-like shape. In line with this hypothesis CDH9, which is up-regulated during EMT (epithelial to mesenchymal cell transition) of renal tubular epithelial cells [37], and CDH12, whose overexpression increases the invasive properties of salivary adenoid cystic carcinoma cells [38], are down-regulated in 46BR.1G1 cells. We also analyzed two members of the cadherin family whose expression is commonly used as a diagnostic marker of EMT events: *CDH1* and *CDH2* genes, which are respectively down and up regulated during EMT. The RNA-Seq, but not the microarray analysis, evidenced a moderate but statistically significant reduction of *CDH2* mRNA in 46BR.1G1 cells (LFC = -0.66 p-value = 4x10^-4^) while both methods were unable to predict the behavior of *CDH1* because its expression was too low to be analyzed under the experimental conditions used in this study. In agreement with RNA-Seq data, qRT-PCR analysis evidenced statistically significant down-regulation of *CDH2* in LigI-deficient cells accompanied by a slight increase of *CDH1* mRNA (Fig 4, panel B). In particular, CDH2 expression was reduced to about 50% in 46BR.1G1 cells, consistent with the difference estimated by RNA-Seq analysis. The differential expression between 7A3 and 46BR.1G1 of different cadherins is notable. It has been shown that the expression of several cadherin genes is differentially affected by epithelial as opposed to the mesenchymal phenotype. Within this framework, for example CDH9 and CDH12 are up regulated as expected in the more mesenchymal-like line 7A3. CDH1, the prototypical epithelial junctional protein, is elevated in LigI-deficient cells while CDH2 (the mesenchymal N-cadherin) is down regulated. The functional phenotypic consequences of other cadherins is less understood and would be interesting in future to explore their impact on the nature of epithelial vs mesenchymal phenotype. Altogether this analysis is consistent with the idea, suggested by the morphological data, that LigI deficiency induces a shift toward an epithelial-like morphology. Moreover, in agreement with the increase in adhesion properties (Fig 2), the vinculin (*VCL*) gene, which encodes a focal adhesion protein [39], is up-regulated in 46BR.1G1 cells (Fig 4 panel C). Up-regulation of vinculin was detected only by the micro-array and confirmed by qRT-PCR but not by the RNA-Seq analysis, once more pointing to the cautions that must be put in the interpretation of genome wide data, particularly when low number of reads are considered in RNA-Seq experiments. We also evaluated the expression of vimentin (*VIM*) a member of the intermediate filaments family of proteins responsible for maintaining cell shape, and whose expression is typically up regulated during EMT. In accord with microarray and RNA-Seq data, qPCR analysis detected a comparable expression of vimentin in 46BR.1G1 and 7A3 cells (Fig 4 panel C).

Since morphometric parameters of 46BR.1G1 cells become similar to those of 7A3 cells upon ATM inhibition, we investigated whether expression level of the genes discussed above could be affected by KU-55933, a specific ATM inhibitor. As shown in Fig 4, treatment with KU-55933 significantly decreases the levels of *CDH13 (*P = 0.0054), *CDH4 (*P = 0.0386), and vinculin (*VCL* P = 0.0331) mRNAs (panel A and C) only in 46BR.1G1 cells where they are up regulated. In spite of a similar trend, treatment with KU-55933 in 7A3 cells did not show statistically significant difference in the expression levels of the analyzed genes. On the contrary, the drug has no significant effect on *CDH1* gene (P = 0.4735), up regulated in 46BR.1G1, and on *CDH9* (P = 0.7173), *CDH12* (P = 0.7609) and *CDH2* (P = 0.4735) which are more expressed in 7A3 cells, suggesting the existence of additional levels of complexity in controlling gene expression regulation in response to DNA damage in 46BR.1G1 cells. Collectively, our analysis indicates that replication-dependent DNA damage may affect the expression level of a number of genes involved in cytoskeletal organization through the activation of kinases of the checkpoint pathways, in agreement with the hypothesis that DDR programs impact on cell morphology and motility processes.

## Discussion

LigI-deficient 46BR.1G1 cells represent a good model to investigate the biological effects of sub-lethal levels of DNA insults. Indeed, the cyclic induction of DNA damages in successive S-phases, resulting from a defect in the maturation of the Okazaki fragments, is sufficient to elicit a moderate ATM-dependent DDR that mildly lengthens the cell-cycle without triggering cell apoptosis or senescence [3]. Unexpectedly, LigI-deficiency also perturbs morphological cell features and impacts the organization of stress fibers, a distinctive feature of fibroblasts. In this manuscript we have quantified the morphological and migratory differences between LigI-mutated 46BR.1G1 and their derivatives 7A3 cells in which the replication defect has been rescued by the stable expression of wild type LigI cDNA. During this analysis we have observed that differences between the two cell lines can be greatly reduced by growing 46BR.1G1 cells for 24 hours in the presence of the ATM inhibitor KU-55933, raising the hypothesis that a modest DNA damage response can affect cell phenotype. However, the failure of ATM inhibition to completely revert the phenotype of 46BR.1G1 cells to the fibroblast morphology seems to indicate the involvement of additional mechanisms. It is conceivable that a persistent moderate level of DNA damage may trigger gene expression changes that are resistant to the temporary inhibition of checkpoint kinases, particularly if the source of the damage (i.e. LigI deficiency) is not removed. Only hypothesis can be raised at this moment about the players involved. A plausible candidate is the epigenetic organization. Indeed, DNA repair mechanisms and DNA damage signaling are known to affect chromatin organization and histone post-translational modifications [40]. Whether these marks affect specific gene expression circuits relevant to the morphology of 46BR.1G1 cells is an open question we are presently investigating. Whatever is the mechanism involved in this phenomenon, we speculate that such an effect of moderate DNA damage may be physiologically relevant during developmental and cell differentiation programs or may play a role in a number of pathological conditions such as cancer and some neurological disorders, as for instance Parkinson’s or Alzheimer’s disease. Although highly hypothetical, our proposal is in line with a number of observations. Thus, a DNA damaging agent like hypoxia plays a role in developmental programs [41,42], metastatic dissemination of cancer cells [43] and neurological disorders [44]. Moreover it has been recently observed that DNA damage drives differentiation of leukemic cells [45]. Another example is the signaling pathway identified by p38 and MAPKAP kinase-2 (p38/MK2) that operates in the cytoplasm downstream of ATM and ATR. p38/MK2 can affect cell biology by modulating the stability of mRNAs containing AU-rich elements in their 3’-UTR [46].

In order to gain insight into the regulatory circuits underlying the distinctive morphological features of 46BR.1G1 cells in response to replicative DNA damage, we have compared the gene expression profiles in 46BR.1G1 and 7A3 by means of two genome wide approaches, namely microarrays and RNA-Seq. The results of these analyses raise two types of considerations. One is methodological and concerns the reciprocal validation of the two assays. We have observed only a partial overlapping between the lists of genes selected by the two approaches (2114 by the microarray and 855 by RNA-Seq). This may partially originate from the limited number of reads (40 millions) used in the RNA-Seq analysis. However, it also emphasizes the caution in comparing data produced with different genome-wide approaches, a problem already discussed in a recent publication [47]. On the other hand, the differences between the two methods are almost completely eliminated when, instead of single genes, bio-functional categories selected by the IPA Ingenuity program are taken into account. Notably the same list of categories account for most of the 375 genes (corresponding to 43.9% of the RNA-Seq data) that form the common core of differentially expressed gene identified both by RNA-Seq and microarray analysis.

The second consideration pertains the concordance between the bio-functional categories and the cell morphology and migration properties evidenced by our functional assays. Interestingly, all our data seem to indicate that LigI-deficiency can promote a transition of fibroblasts toward an epithelial phenotype both in term of cell morphology, migration properties and gene expression profiles. The regulatory circuits acting downstream of ATM and involved in this transition are still matter of investigation. Recently, a number of transcriptional regulators have been shown to be targets of checkpoint signaling kinases ATM and ATR [23,30,31]. This list includes 14 transcription factors that are predicted by the IPA analysis as upstream regulators of genes differentially expressed in 46BR.1G1 vs 7A3 cells and highly enriched in IPA biological categories related to cytoskeletal-based functions (S4 Table). The identification of the regulatory circuits underlying this DNA damage-induced transition will open new perspectives to the analysis of cell differentiation programs.

## Supporting Information

S1 Fig Bright-field microscopy of 46BR.1G1 and 31W cells. (TIF)

S2 Fig Parameters of cell migration. A) Accumulated distance, B) Velocity, C) Directionality were calculated from analysis of 16 cells in 3 independent experiments. Bars show mean ± SEM. The analysis was performed by Chemotaxis and Migration plug-in for Image J software (version 1.01) distributed by Ibidi.
(TIF)

S1 Table Primers for real time RT-PCR. (DOC)

S2 Table IPA Core Analysis. (XLS)

S3 Table Complete list of the genes alternative expressed. (XLS)

S4 Table Upstream regulators. (XLS)

S1 Video Control fibroblasts. (MOV)

S2 Video 7A3 cells. (MOV)

S3 Video 46BR.1G1 cells. (MOV)
