# Supplementary figures and images for: Chronic Replication Problems Impact Cell Morphology and Adhesion of DNA Ligase I Defective Cells
Source: PLoS One. 2015 Jul 7;10(7):e0130561. doi: 10.1371/journal.pone.0130561 (PMC4495043; doi:10.1371/journal.pone.0130561)

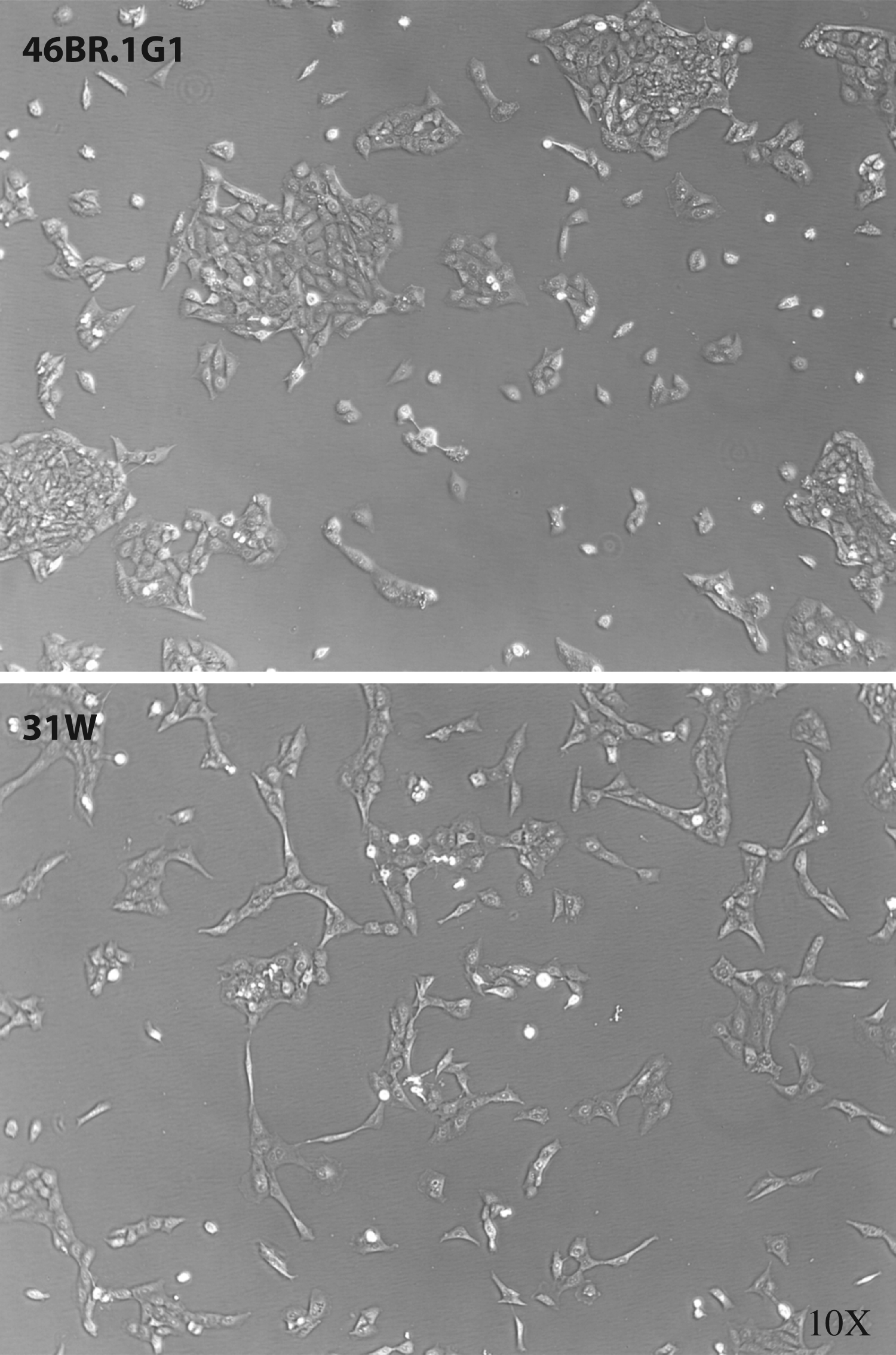

Supplement: S1 Fig — (TIF) [file pone.0130561.s001.tif]

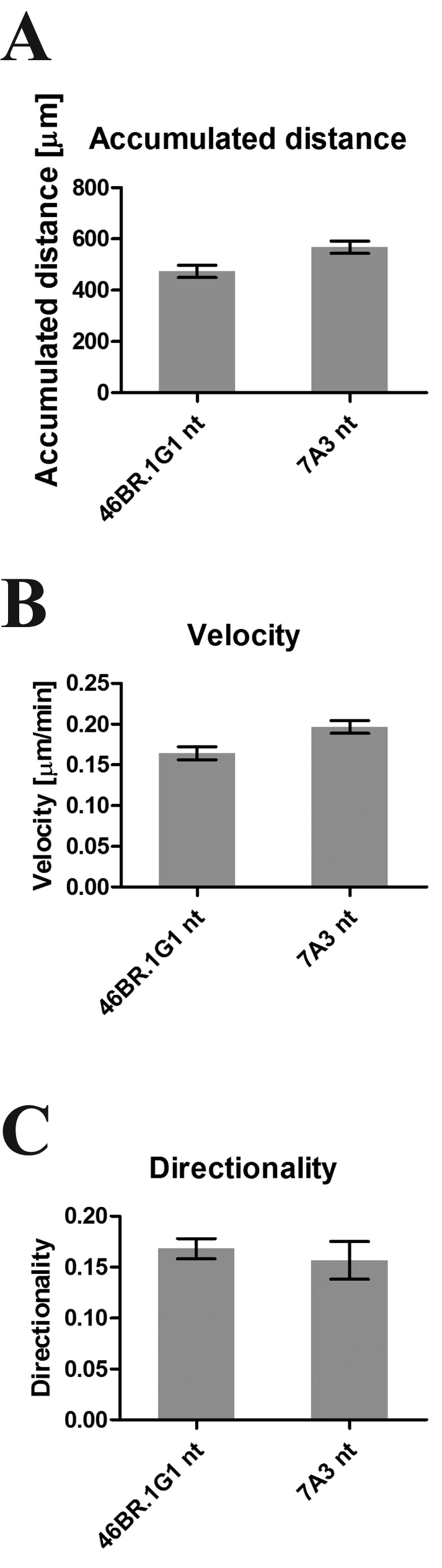

Supplement: S2 Fig — A) Accumulated distance, B) Velocity, C) Directionality were calculated from analysis of 16 cells in 3 independent experiments. Bars show mean ± SEM. The analysis was performed by Chemotaxis and Migration plug-in for Image J software (version 1.01) distributed by Ibidi. (TIF) [file pone.0130561.s002.tif]
