# Supplementary material for: Chronic Replication Problems Impact Cell Morphology and Adhesion of DNA Ligase I Defective Cells
Source: PLoS One. 2015 Jul 7;10(7):e0130561. doi: 10.1371/journal.pone.0130561 (PMC4495043; doi:10.1371/journal.pone.0130561)
Supplement: S1 Table — (DOC) [file pone.0130561.s003.doc]

**S1 Table: primers for real time RT-PCR**

| Oligonucleotide name | Sequence 5’ |
| --- | --- |
| CDH1_Fw | CTGGGACTCCACCTACAGAAAGTT |
| CDH1_Rev | GAGGAGTTGGGAAATGTGAGCA |
| CDH12_Fw | CCGCAGGCAGCAAGAGTTGTATTT |
| CDH12_Rev | ACAAGACAGGATGGTGCCATCAGA |
| CDH13_Fw | TGTACACTGCTCTCTTCCTGGCAA |
| CDH13_Rev | ATTGTCATTCACGTCCTCCAGGGT |
| CDH2_Fw | TTCCTGAGGGATCAAAGCCTGGAA |
| CDH2_Rev | TACCTCAACATCCCATTGAGGGCA |
| CDH4_Fw | TTCACCATCAACAGCGAGACTGGA |
| CDH4_Rev | ATCTGTGGCCTGAACGATGACTGT |
| CDH9_Fw | ACCGGGAATCATCTCCTTGGCATA |
| CDH9_Rev | GGCAAATTCCGGAGCATGGTCATT |
| RPLP0_Fw | ATGCCCAGGGAAGACAGGGCG |
| RPLP0_Rev | CGAAGGGACATGCGGATCTGCTGC |
| VCL_Fw | AATTCCGAGGATCCCAAGTTCCGT |
| VCL_Rev | CGGAAATGTTTCCAGCCACAGCTT |
| Vimentin_Fw | GTGGACCAGCTAACCAACGACAAA |
| Vimentin_Rev | TTCAAGGTCAAGACGTGCCAGAGA |
